# Supplementary material for: Day-to-day spontaneous social behaviours is quantitatively and qualitatively affected in a 16p11.2 deletion mouse model
Source: Front Behav Neurosci. 2023 Dec 19;17:1294558. doi: 10.3389/fnbeh.2023.1294558 (PMC10763239; doi:10.3389/fnbeh.2023.1294558)
Supplement: DATA SHEET 2 — Availability of raw data and python scripts to process data. [file Data_Sheet_2.pdf]

Quartets of 4 mice:

Males:

M1: https://doi.org/10.5281/zenodo.7414261

M2: https://doi.org/10.5281/zenodo.7413572

M3: https://doi.org/10.5281/zenodo.7413803

M4: https://doi.org/10.5281/zenodo.7413878

M6: https://doi.org/10.5281/zenodo.7414252

M7: https://doi.org/10.5281/zenodo.7414261

Females:

F1: https://doi.org/10.5281/zenodo.7414941

F2: https://doi.org/10.5281/zenodo.7415307

F3: https://doi.org/10.5281/zenodo.7418326

F4: https://doi.org/10.5281/zenodo.7418500

F5: https://doi.org/10.5281/zenodo.7418630

F7: https://doi.org/10.5281/zenodo.7418870

F9: https://doi.org/10.5281/zenodo.7419017

F10: https://doi.org/10.5281/zenodo.7419023

Pairs of mice:

5170_5256: https://doi.org/10.5281/zenodo.7408169

5160_5182: https://doi.org/10.5281/zenodo.7408502

5186_5172: https://doi.org/10.5281/zenodo.7408709

5194_5231: https://doi.org/10.5281/zenodo.7409004

5220_5175: https://doi.org/10.5281/zenodo.7409156

5257_5180: https://doi.org/10.5281/zenodo.7409410

5195_5167: https://doi.org/10.5281/zenodo.7409521

5197_5212: https://doi.org/10.5281/zenodo.7409665

5202_5191: https://doi.org/10.5281/zenodo.7409989

5187_5230: https://doi.org/10.5281/zenodo.7410224

5196_5050: https://doi.org/10.5281/zenodo.7410354

5171_5164: https://doi.org/10.5281/zenodo.7410504

5206_5188: https://doi.org/10.5281/zenodo.7410508

5185_5165: https://doi.org/10.5281/zenodo.7410514

5163_5199: https://doi.org/10.5281/zenodo.7411821

5224_5173: https://doi.org/10.5281/zenodo.7411827

5189_5193: https://doi.org/10.5281/zenodo.7411830

5169_5208: https://doi.org/10.5281/zenodo.7411835

Pipeline for data analyses:

https://github.com/fdechaumont/lmt-analysis

Quartets of mice:

0. Recontruct the behavioural events: lmt-analysis>LMT>scripts>Rebuild_All_Events.py

1. Generate the json file to store data for the behavioural profiles: lmt-analysis>LMT>scripts>ComputeMeasuresIdentityProfileOneMouseAutomatic.py [1] (either on the first 15 min or over the three nights)

2. Generate the json file to store data for the selective interactions: lmt-analysis>LMT>scripts>ComputeMeasuresIdentityProfileOneMouseAutomaticPerIndividual.py [1] (either on the first 15 min or over the three nights)

Pairs of mice:

0. Recontruct the behavioural events: lmt-analysis>LMT>scripts>Rebuild_All_Events.py

1. Generate the json file to store data for the behavioural profiles: lmt-analysis>LMT>scripts>ComputeMeasuresIdentityProfileOneMouseAutomatic.py [2] (either on the first 15 min or over the three nights)

2. Generate the json file to store data for the usage of ultrasonic vocalisations: USV-analysis>LMT>USV>Compute_Number_USVs_Diff_Geno.py [1] and [1a]

3. Generate the json file to store data for the correlation of USVs with behavioural events with either USVs or behaviours as references: USV-analysis>LMT>USV>Compute_Number_USVs_Diff_Geno.py [2] and [2a]

4. Generate the json file to store data for acoustic features of USVs: USV-analysis>LMT>USV>Compute_Number_USVs_Diff_Geno.py [3] and [3a]

5. Reconstruct exclusive behavioural events: lmt-analysis>LMT>scripts>Rebuild_All_Exclusive_Contact_Events.py

6. Compute and analyse the transitions between exclusive events: lmt-analysis>LMT>scripts>BehaviouralSequences>ComputeTransitionsBetweenEvents.py
